# The Use of Expert Opinion to Assess the Risk of Emergence or Re-Emergence of Infectious Diseases in Canada Associated with Climate Change

**DOI:** 10.1371/journal.pone.0041590

**Published:** 2012-07-27

**Authors:** Ruth Cox, Crawford W. Revie, Javier Sanchez

**Affiliations:** Atlantic Veterinary College, University of Prince Edward Island, Charlottetown, Prince Edward Island, Canada; University of Ottawa, Canada

## Abstract

Global climate change is predicted to lead to an increase in infectious disease outbreaks. Reliable surveillance for diseases that are most likely to emerge is required, and given limited resources, policy decision makers need rational methods with which to prioritise pathogen threats. Here expert opinion was collected to determine what criteria could be used to prioritise diseases according to the likelihood of emergence in response to climate change and according to their impact. We identified a total of 40 criteria that might be used for this purpose in the Canadian context. The opinion of 64 experts from academic, government and independent backgrounds was collected to determine the importance of the criteria. A weight was calculated for each criterion based on the expert opinion. The five that were considered most influential on disease emergence or impact were: potential economic impact, severity of disease in the general human population, human case fatality rate, the type of climate that the pathogen can tolerate and the current climatic conditions in Canada. There was effective consensus about the influence of some criteria among participants, while for others there was considerable variation. The specific climate criteria that were most likely to influence disease emergence were: an annual increase in temperature, an increase in summer temperature, an increase in summer precipitation and to a lesser extent an increase in winter temperature. These climate variables were considered to be most influential on vector-borne diseases and on food and water-borne diseases. Opinion about the influence of climate on air-borne diseases and diseases spread by direct/indirect contact were more variable. The impact of emerging diseases on the human population was deemed more important than the impact on animal populations.

## Introduction

It has been predicted that “global warming” will cause unprecedented changes to the earth's climate [1]. North America and the Arctic regions in particular, will experience warmer temperatures, more rainfall, more frequent droughts, and extreme weather events such as hurricanes and tornadoes [2]–[4]. These events are likely to change the incidence and distribution of emerging and re-emerging infectious diseases [1]. Climate could affect, for example, the range and population size of pathogens, hosts and vectors, the length of the transmission season, and the timing and persistence of outbreaks. Increased temperatures and altered rainfall patterns are expected to promote the geographic occurrence and abundance of vector-borne and water-borne diseases in particular [1]. Changes in climate might also hinder the emergence of some diseases. Milder winters and hotter, humid summers could favour West Nile virus and Lyme disease but drought or heavy rainfall may keep them in control [5]. Finally, the distribution of diseases may be indirectly affected, as the impacts of climate change in some areas (desertification, flooding) may lead to migration of human populations and changes in human behaviour [3].

The impact of disease emergence in Canada could be substantial. The economic effects of zoonoses in Canada range from lost work productivity to international trade and travel restrictions [6]. The economic cost of gastro-intestinal illness in Canada, for example, has been estimated at $3.7 billion annually [7].

In order to detect and respond to future disease agents emerging as a result of climate change, reliable surveillance for diseases that are most likely to be influenced by climate is required, with particular attention to those with potentially large public health impacts [8]. Policy decision makers therefore need to be able to prioritise a list of potential emerging or re-emerging pathogens that are likely to affect animal and human populations. We define an emerging infectious disease as a disease that has newly appeared in a population or that has existed but is rapidly increasing in incidence or geographic range [9].

Rational priority setting requires understanding of a complex system, since different criteria and priorities will affect the decision to address a particular disease threat. In recent years progress has been made in identifying the key characteristics of potential emerging infectious diseases and attempts have been made to prioritise infectious diseases in terms of their risk of emergence or impact in some countries (e.g.[10], [11]). Here the focus is on pathogen characteristics and the potential influence of climate change in Canada.

The aim of this work was to identify criteria that might be used to prioritise diseases and to use expert opinion to determine the importance of these criteria. Expert opinion was collected via electronic questionnaire. Collation of this expert opinion is essential to subsequent work that will develop ranking methods and multi-criterion decision approaches that can be used to prioritise human and animal disease threats according to how likely they are to emerge in Canada in response to climate change and according to their impact.

## Methods

### Ethics statement

The study protocol including the written consent of all participants, was approved by the University of Prince Edward Island Research Ethics Board (REB Reference #6003938).

### Study design summary

Identification of criteria and elicitation of expert opinion followed the procedure outlined by [12], which draws on several existing protocols. It involved the following steps:

- Questionnaire design, including identification of criteria that might be used to prioritise diseases according to how likely they are to emerge in Canada in response to climate change and according to their impact.
- Recruitment of expert participants and distribution of questionnaire.
- Calculation of criteria weighting based on expert opinion.

### Identification of criteria and questionnaire design

A questionnaire was designed using Microsoft Word 2007 and could be completed electronically. It presented a list of 40 criteria that might be used to prioritise diseases according to how likely they are to emerge in Canada in response to climate change and according to their impact (Table 1). Criteria were identified from published literature, discussion with experts from universities and government agencies, and where possible, informed by previous disease prioritisation work (e.g. [1], [10], [13]–[18]. Some criteria were frequently used in other prioritisation studies (e.g. incidence, severity, mortality), while others were included specifically for the project focus (e.g. criteria related to climate change). A measurement scale was developed for each criterion, although it was not presented to participants in this questionnaire and will not be presented here since the aim of this paper focuses on expert opinion around the criteria.

**Table 1:** List of criteria to prioritise diseases according to how likely they are to emerge in Canada in response to climate change (groups A to C) and according to their impact (groups D and E).

**A. DISEASE EPIDEMIOLOGY**
A1. Pathogen taxonomic group (bacteria, virus, fungi, helminth, protozoa).
A2. Pathogen zoonotic potential (zoonotic or not zoonotic).
A3. Pathogen endemicity to Canada (exotic, introduced sporadically or endemic to Canada).
A4. Current incidence of human disease in Canada (average number of new cases in the last 5 years).
A5. Current incidence of animal disease in Canada (average number of new cases in the last 5 years).
A6. Trend of human disease incidence in Canada in the last 5 years (decreasing, stable or increasing).
A7. Trend of animal disease incidence in Canada in the last 5 years (decreasing, stable or increasing).
A8. Number of ways the pathogen may enter Canada (e.g. via imports, bird migration, human entry).
A9. Type of climate that the pathogen can tolerate (dry, tropical, temperate or continental).
A10. Geographic proximity of the pathogen to Canada.
A11. Mode of transmission (direct, indirect via environmental reservoir or vector-borne).
A12. Amount of information that is known about risk factors for introduction and transmission.
**B. ABILITY TO MONITOR, TREAT AND CONTROL DISEASE**
B1. Effectiveness of national and international surveillance.
B2. Ability to diagnose disease in Canada (availability and sensitivity of diagnostic tests).
B3. Ability to prevent disease in Canada (e.g. by vaccination or public health education).
B4. Ability to treat disease in humans in Canada (availability and effectiveness of treatment).
B5. Ability to treat disease in domesticated animals in Canada (availability and effectiveness of treatment).
**C. INFLUENCE OF CLIMATE CHANGE**
C1. Climatic conditions in Canada.
C2. Presence of definitive host species in Canada.
C3. Annual increase in temperature in Canada.
C4. An increase in summer temperature in Canada.
C5. An increase in winter temperature in Canada.
C6. A decrease in summer temperature in Canada.
C7. A decrease in winter temperature in Canada.
C8. An increase in summer precipitation in Canada.
C9. An increase in winter precipitation in Canada.
C10. A decrease in summer precipitation in Canada.
C11. A decrease in winter precipitation in Canada.
C12. Presence of a suitable vector in Canada.
**D. BURDEN OF DISEASE**
D1. Likely incidence of human disease in Canada
D2. Pathogenicity in the general human population (not pathogenic or frequently pathogenic).
D3. Severity of disease in the general human population (mild, moderate or severe).
D4. Human case fatality rate.
D5. Likely incidence of disease in domesticated animals.
D6. Pathogenicity in domesticated animals (not pathogenic or frequently pathogenic).
D7. Severity of disease in domesticated animals (mild, moderate or severe).
D8. Domesticated animal case fatality rate (including the need for culling).
**E. ECONOMIC, ENVIRONMENTAL AND SOCIAL IMPACT**
E1. Potential economic impact (e.g. cost to industry and for control, health care, travel restrictions).
E2. Potential environmental impact (e.g. impact on air, water, soil, landscape and biodiversity).
E3. Potential social impact (e.g. level of media coverage, level of anxiety of the general population).

To provide structural cohesion, criteria were divided into 5 groups:

#### Section A: Disease epidemiology (containing 12 criteria)

This section focused on pathogen characteristics (criteria A1–A3) and recent trends in disease incidence (A4–A7). Criteria also consider pathogen endemicity and potential for introduction to and transmission within Canada (A8–A12).

#### Section B: Ability to monitor, treat and control disease (5 criteria)

Criteria relate to national and international surveillance (B1), diagnosis (B2), preventability (B3) and treatability (B4–5) in humans and domesticated animals.

#### Section C: Influence of climate (12 criteria)

Criteria consider whether the pathogen, host and vector (where applicable) would be able to survive in the current climate in Canada (C1, C2, C12). The remaining criteria (C3–C11) consider how changes in temperature and precipitation (in summer and winter) might promote or inhibit the emergence of a pathogen. While there are a wide range of climatic factors that might influence pathogen emergence, criteria focused on these two aspects for two reasons. Firstly, the number of criteria needed to be manageable for prioritisation purposes. Secondly these climate processes are well documented compared to others and of significant concern [3]. Global average temperature could rise by 1.4–5.8°C between 1990 and 2100, although Canada is likely to experience greater rates of warming than many other regions of the world [19]. Although climate change may affect different regions of Canada in different ways, details of specific geographic areas were not included at this stage of the prioritisation. Certain types of disease are more likely to be influenced by climate than others, therefore four broad disease groups were identified based on mode of transmission and Section C was repeated for each transmission mode. These criteria will be referred to with a subscript: vector-borne (C_V_), food and water-borne (C_FW_), airborne (C_A_) and direct/indirect contact (C_D_). There were therefore a total of 45 criteria related to climate (12 for vector-borne disease and 11 each for the other modes of transmission. Criterion C_V_12 ‘presence of a suitable vector in Canada’ was only relevant to vector borne disease).

#### Section D: Burden of disease (8 criteria)

While the aim of this work was not a formal risk assessment, sections D and E considered likely impacts if a pathogen did emerge in Canada. Section D included criteria about disease incidence, pathogenicity, severity and fatality in the human population and in the domesticated animal population. Domesticated animal population was specified because estimation of burden in wildlife populations was beyond the scope of this research.

#### Section E: Economic, environmental and social impact (3 criteria)

This section included three criteria to capture information about economic impact (E1 - including costs for control and health care), environmental impact (E2 - including the impact on the environment and biodiversity) and social impact (E3 - including the perception in the media and general population) of disease emergence in Canada.

In sections A, B and C questions were posed in the form: ‘Is *this criterion* likely to influence the *probability* of an infectious disease emerging in Canada?’. Participants were asked to select one answer from: ‘don't know’, ‘not likely’, ‘quite likely’, ‘likely’, ‘very likely’ or ‘extremely likely’. In sections D and E participants were asked, ‘How important is *this criterion* for prioritising infectious diseases in terms of their *impact* if they did emerge in Canada in response to climate change?’. One answer could be selected from the options: ‘don't know’, ‘not important’, ‘quite important’, ‘important’, ‘very important’, and ‘extremely important’. Cross boxes were provided for responses.

Experts were provided with the following example as guidance: ‘if you think that one or some pathogen taxonomic groups are more likely to emerge in response to climate change than others then you think that this criterion is likely to influence the probability of a disease emerging in response to climate change. Therefore cross one of the boxes labelled ‘quite likely’, ‘likely’, ‘very likely’ or ‘extremely likely’ as appropriate. If you think that the pathogen taxonomic group does not influence whether a disease will emerge in response to climate change then cross the ‘not likely’ box’. Selection of the ‘don't know’ answer was at the discretion of the expert, however it was made clear that the start of the questionnaire that the aim was to collect opinion, not to assess level of knowledge. Participants were invited to suggest other criteria or to alter existing criteria and space for additional written information was provided.

At the end of the questionnaire two additional questions were presented. Experts were asked to rank (in ascending order from 1 to 5) the following taxonomic groups: bacteria (including rickettsia), viruses (including prions), protozoa, fungi and helminths, according to how likely they are to be influenced by climate (5 = most likely to be influenced by climate). The same rank could be chosen more than once if required. Similarly participants were asked to rank the following modes of transmission: direct/indirect contact, air-borne, water-borne, food borne and vector-borne. Broad disease groupings were proposed based on [1] and were modified following discussions with experts during a pre-test phase.

The questionnaire was pre-tested by individuals at the University of Prince Edward Island (UPEI), the Public Health Agency of Canada (PHAC) and Canadian Food Inspection Agency before being distributed to all participants.

### Recruitment of expert participants and distribution of questionnaire

Experts in the areas of infectious disease epidemiology, in particular emerging diseases and climate change, were identified through literature and internet searching and via recommendations from other recruited participants as well as individuals at PHAC, British Columbia Centres for Disease Control, Ontario Ministry of Agriculture, Veterinary Laboratories Agency UK and Aboriginal Environmental Health. Experts were defined as individuals whose past or present field contains the subject under study i.e. infectious disease epidemiology and/or climate change, following [20]. Inclusion criteria followed recommendations by [21], which include evidence of expertise, understanding of the problem area, reputation, availability and willingness to participate.

Experts were invited to take part in the research via an email that explained the aim, methods and use of study data. The questionnaire was approved by UPEI's Research Ethics Board and the information included a page for written informed consent. The aim was to include individuals from a variety of backgrounds, including public and animal health, federal and provincial agencies, universities and independent organisations. The majority of participants were based in Canada.

The questionnaire, project summary and instructions were emailed to participants who were asked to respond within 10 days. Non-responders were reminded once after 2 to 3 weeks. A Delphi-like approach was used to obtain feedback from the participants at the end of the study [22]. This involved providing an anonymous summary of group results to participants and inviting them to review and revise their individual answers in light of the response from the other experts if they wished.

### Calculation of criteria weighting based on expert opinion

Expert responses were used to calculate a weight for each of the criteria and therefore to determine a relative ranking of criteria. The ‘likelihood’ score (sections A, B and C) and the ‘importance’ score (sections D and E) was converted to values of 0, 0.1, 0.3, 0.5, 0.7, 0.9 and a weight calculated as the mean score of all experts. This linear scale was chosen following methods used by [10]. The number of ‘don't know’ responses per criteria was tabulated as an indicator of the level of uncertainty. These responses were excluded from the mean weight calculation. (Throughout ‘uncertainty’ will be used to describe ‘don't know’ responses, compared to ‘variability’ which will be used to describe the range of expert response).

## Results

### Expert response

Of the 121 experts invited to take part (Table 2) a total of 86 agreed to contribute to this work. Of the remainder, 22 individuals did not reply to the email, while 13 declined due to lack of expertise or time. Of the 86, 64 completed the questionnaire (response rate of 74%). Most respondents (55) were based at Canadian institutions, others resided in the USA (4), UK (3), France (1) and Japan (1), although most had specific knowledge of epidemiology in Canada.

**Table 2:** Affiliation of participants and response rates.

Affiliation	Invited to contribute	Completed questionnaire
Government	39	19
Provincial government	22	14
Academic	50	27
Academic and government	4	1
Independent	6	3
Total	121	64

### Revision of Expert Responses

A total of 19 participants responded after being sent an anonymous summary of group results. Of these, 15 participants did not alter any responses, while four altered their responses to an average of 7 criteria (out of 73). The majority of participants chose not to respond during this phase.

### Summary of expert opinion

The ten criteria that were considered to be most likely to influence the probability of a pathogen emerging in response to climate change, or the impact if the pathogen did emerge in Canada, were topped by potential economic impact and severity and fatality of disease in the general human population (Table 3). In the following summary the overall rank of the criterion (of a total of 73) is indicated in brackets.

**Table 3:** Ten criteria deemed the most likely to influence pathogen emergence or impact.

Criteria		Weighting
**E1**	Potential economic impact	0.713
**D3**	Severity of disease in the general human population	0.710
**D4**	Human case fatality rate	0.710
**A9**	Type of climate that the pathogen can tolerate	0.707
**D1**	Likely incidence of human disease in Canada	0.697
**C1**	Climatic conditions in Canada	0.697
**C12**	Presence of a suitable vector in Canada	0.697
**C2**	Presence of definitive host species in Canada	0.683
**A11**	Mode of transmission	0.660
**D2**	Pathogenicity in the general human population	0.654

Criteria labelled A to C relate to emergence, criteria labelled D and E relate to impact. The weight was calculated as the mean score of all participant responses.

#### Disease epidemiology

Most criteria relating to disease epidemiology were deemed likely to influence the probability of disease emergence, in particular the type of climate that the pathogen can tolerate (4), the mode of transmission (9) and the number of ways that the pathogen can enter Canada (19).

#### Ability to treat and prevent disease

Of the prevention criteria, the effectiveness of national and international surveillance (15) was considered most important when prioritising pathogen emergence. The ability to diagnose (20) and prevent (24) disease was more likely to influence disease emergence than the ability to treat (41) disease in Canada.

#### Influence of climate change in Canada

The two criteria that were most likely to influence a disease emerging in Canada were current climatic conditions in Canada (6), and the presence of a definitive host species in Canada (8). These criteria always ranked the highest irrespective of type of disease within this group (Table 4). The presence of a suitable vector also ranked very highly for vector-borne diseases. Changes in climate that tended to be of most concern were an annual increase in temperature, an increase in summer precipitation and an increase in summer temperature.

**Table 4:** Mean weight for criterion describing the influence of climate on disease emergence in Canada.

Climate criteria	Mean Weight			
	Vector-borne	Food and water-borne	Air-borne	Direct/indirect contact
C1 Climatic conditions in Canada	0.697	0.558	0.497	0.432
C2 Presence of a definitive host species in Canada	0.683	0.563	0.526	0.518
C3 An annual increase in temperature in Canada	0.585	0.482	0.369	0.379
C4 An increase in summer temperature in Canada	0.572	0.500	0.369	0.378
C5 An increase in winter temperature in Canada	0.551	0.400	0.343	0.369
C6 A decrease in summer temperature in Canada	0.322	0.247	0.258	0.263
C7 A decrease in winter temperature in Canada	0.314	0.230	0.324	0.263
C8 An increase in summer precipitation in Canada	0.597	0.524	0.329	0.353
C9 An increase in winter precipitation in Canada	0.412	0.409	0.239	0.300
C10 A decrease in summer precipitation in Canada	0.464	0.373	0.304	0.296
C11 A decrease in winter precipitation in Canada	0.323	0.278	0.270	0.227
C12 Presence of a suitable vector in Canada	0.697	NA	NA	NA

The weight was calculated as the mean score of all participant responses for four different modes of pathogen transmission.

#### Burden of disease

The severity of disease (2), human case fatality rate (3) and likely incidence in the human population (5) were of considerable importance. Criteria related to the impact on the animal population were of less concern – animal case fatality rate (17) was of most importance followed by likely incidence in the domestic animal population (23) and severity of animal disease (28).

#### Economic, social and environmental impact

All three criteria in this group were considered ‘very important’ when assessing the impact of an emerging disease. Overall the economic impact (1) was of most concern, the social impact (12) and environmental impact (13) were of less concern.

### Ranking of pathogen taxonomic group and mode of transmission

**Figure 1:**
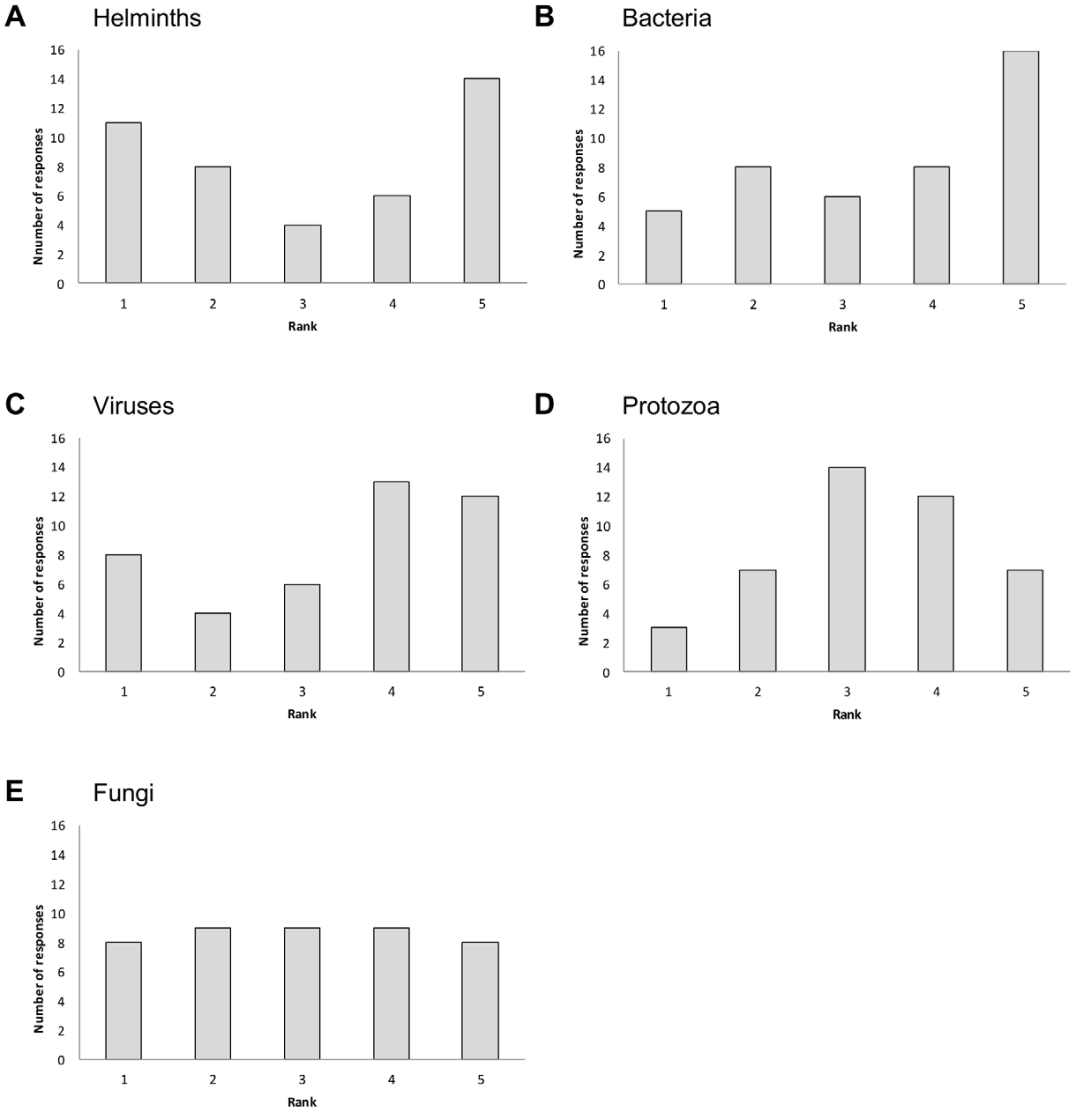
Pathogen taxonomic group ranked by 47 experts according to likelihood of being influenced by climate. a: helminths, b: bacteria, c: viruses, d: protozoa, e: fungi. A rank of 1 indicates least likely and a rank of 5 most likely to be influenced by climate.

A total of 47 participants completed the questions related to ranking. Bacteria were most likely to be influenced by climate (ranking number 5 most often), viruses tended to rank 4 or 5, and protozoa tended to rank 3 (Figure 1). There was more variation in expert opinion about the ranking of fungi and helminths. In particular, there did not appear to be consensus about the influence of climate on helminths, since 13 participants assigned a rank of 5 (most likely to be influenced by climate) – second only to bacteria in this regard - while 11 participants assigned a rank of 1 (least likely to be influenced by climate).

**Figure 2:**
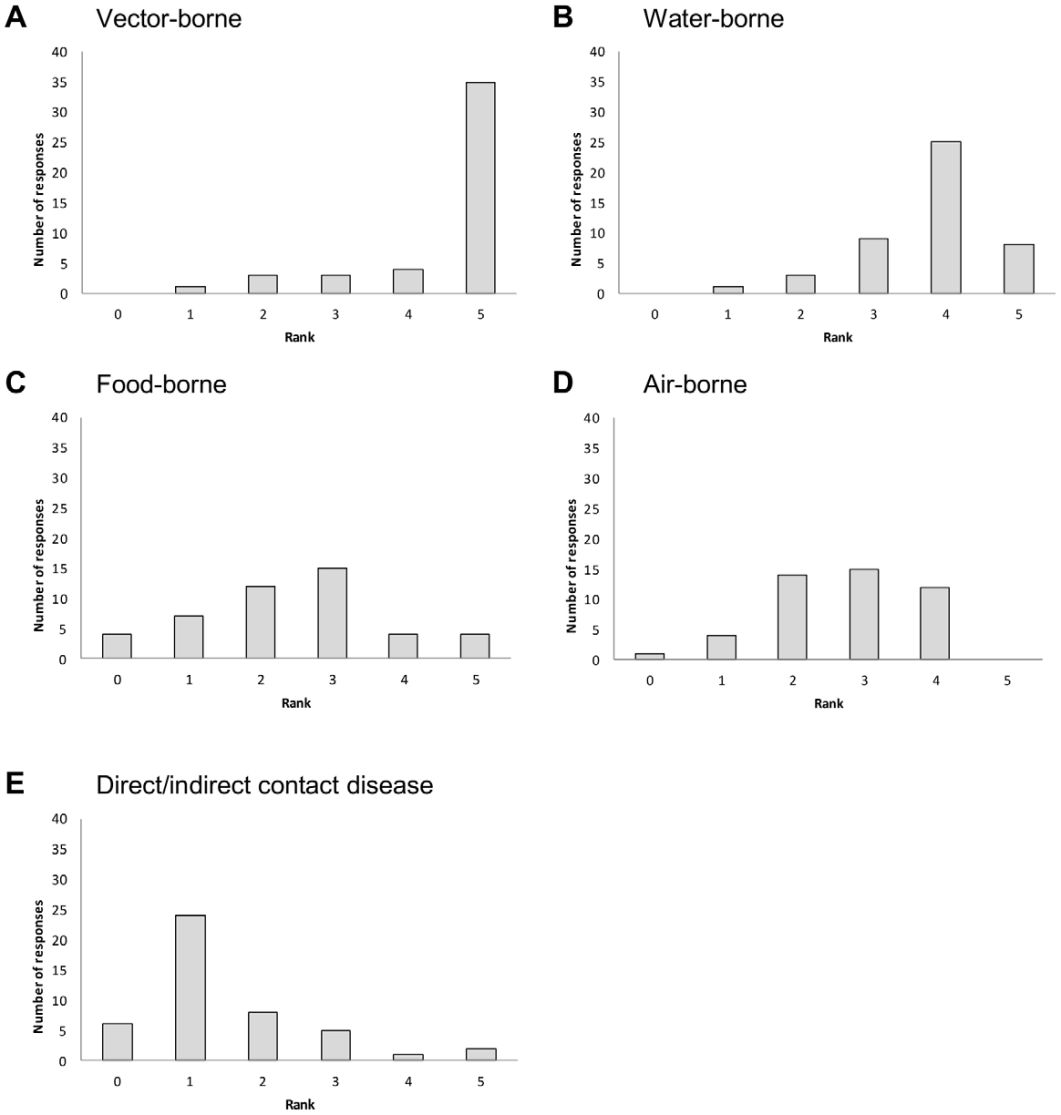
Disease mode of transmission ranked by 45 experts according to likelihood of being influenced by climate. a: vector-borne, b: water-borne; c: food-borne; d: air-borne; e: direct/indirect contact transmission. A rank of 1 indicates least likely and a rank of 5 most likely to be influenced by climate. A score of 0 indicated the opinion that climate does not have an influence on diseases with this mode of transmission.

Participants typically ranked vector-borne diseases as the most likely to be influenced by climate (ranking of 5), water-borne diseases ranked 4, food-borne and air-borne diseases ranked 3, with direct/indirect contact diseases having the lowest rank (Figure 2).

### Variability of expert opinion

**Figure 3:**
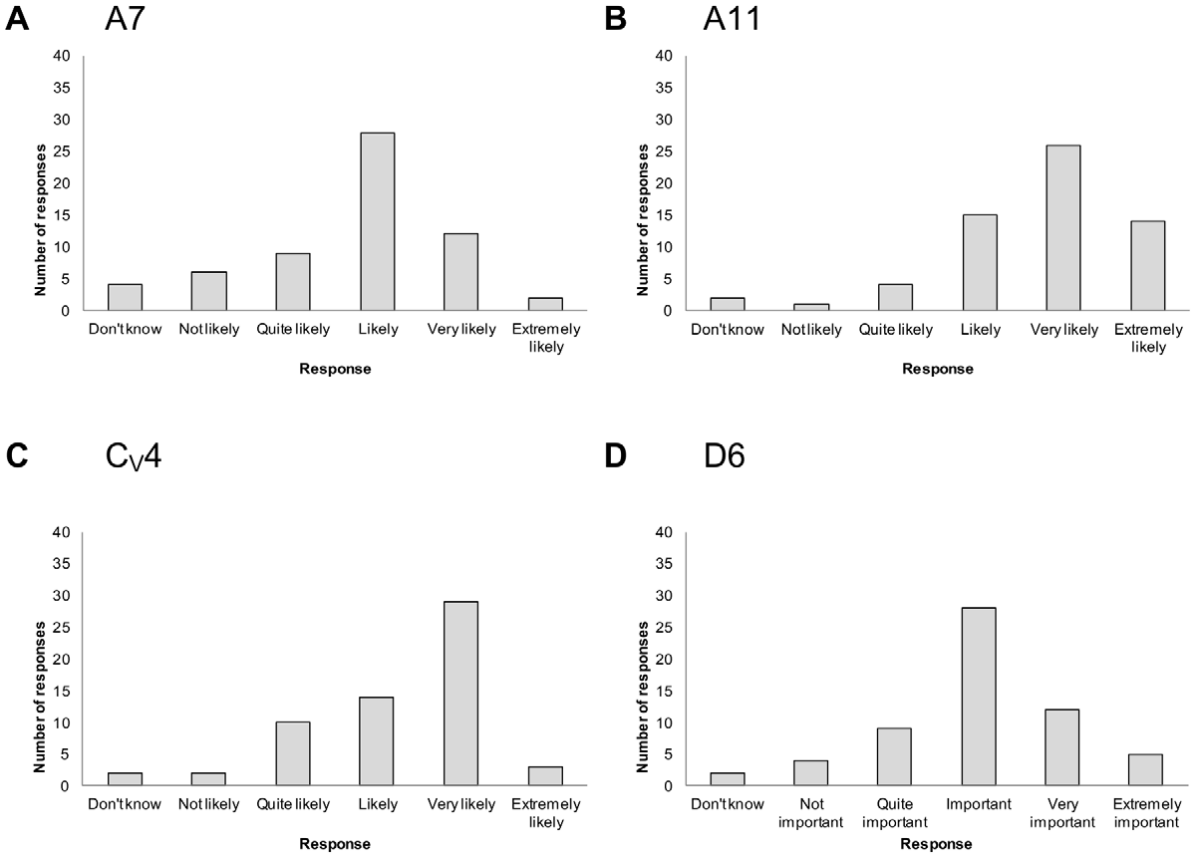
Opinion of 64 experts about the likelihood that ‘*a criterion*’ will influence infectious disease emergence - examples in which participants' opinions were generally in agreement. Criteria (number and description): a: A7 trend of animal disease incidence in Canada in the last five years; b: A11 mode of pathogen transmission; c: C_V_4 an increase in summer temperature in Canada; d: D6 pathogenicity in domesticated animals.

The opinion around some criteria reached a fair degree of agreement. For example, participants generally agreed that trends of animal disease incidence in Canada over the past five years and the mode of transmission are ‘likely’ and ‘very likely’ to influence the probability of an infectious disease emerging in Canada in response to climate change respectively (Figure 3a and 3b). Similarly an increase in summer temperature is ‘very likely’ to influence vector-borne disease emergence and pathogenicity in domesticated animals is an ‘important’ criterion for prioritising infectious diseases in terms of their impact (Figure 3c and 3d respectively).

**Figure 4:**
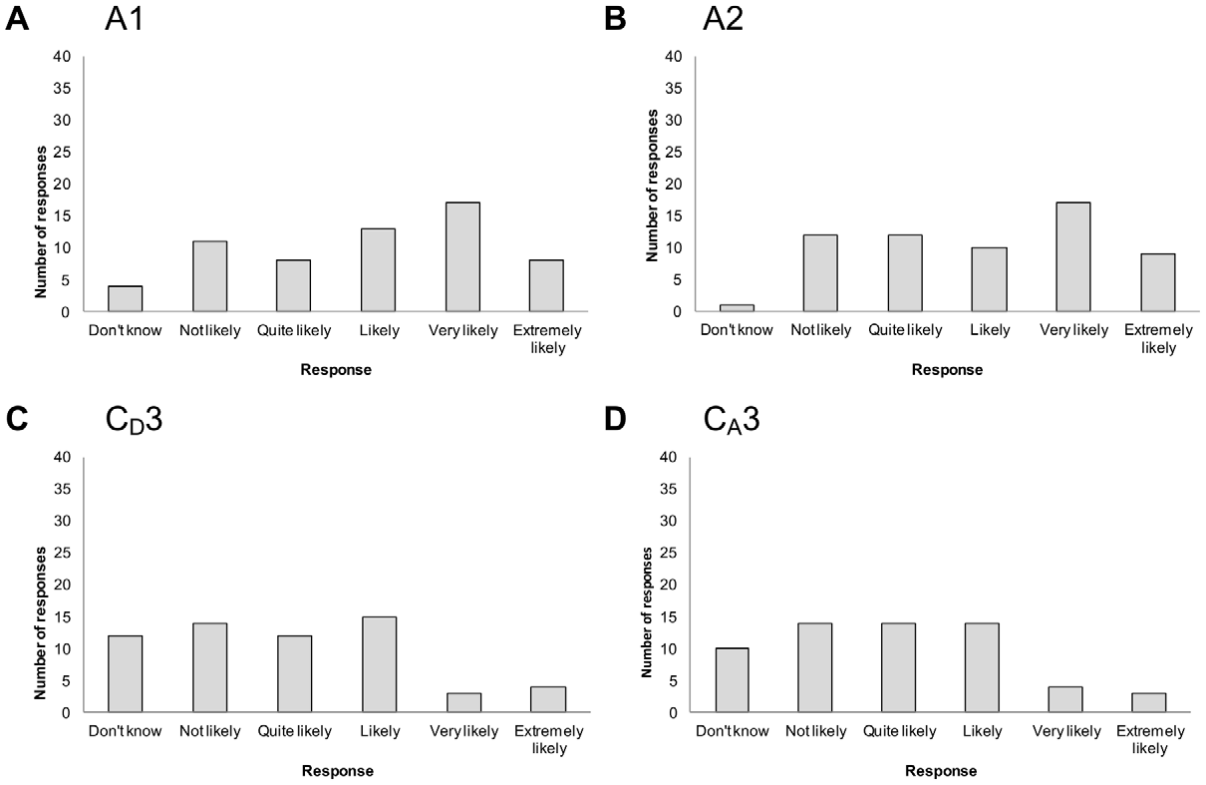
Opinion of 64 experts about the likelihood that ‘*a criterion*’ will influence infectious disease emergence - examples in which participants' opinions were highly variable. Criteria (number and description): a: A1 pathogen taxonomic group; b: A2 pathogen zoonotic potential; c: C_D_3 an annual increase in temperature in Canada; d: C_A_3 an annual increase in temperature in Canada.

In contrast, there appeared to be little agreement among participants for other criteria. For example, responses were highly variable around views on the influence of criteria such as pathogen taxonomic group, pathogen zoonotic potential, an annual increase in temperature on direct/indirect contact diseases, and an annual increase in temperature on air-borne diseases (Figure 4a, b, c and d respectively).

**Figure 5:**
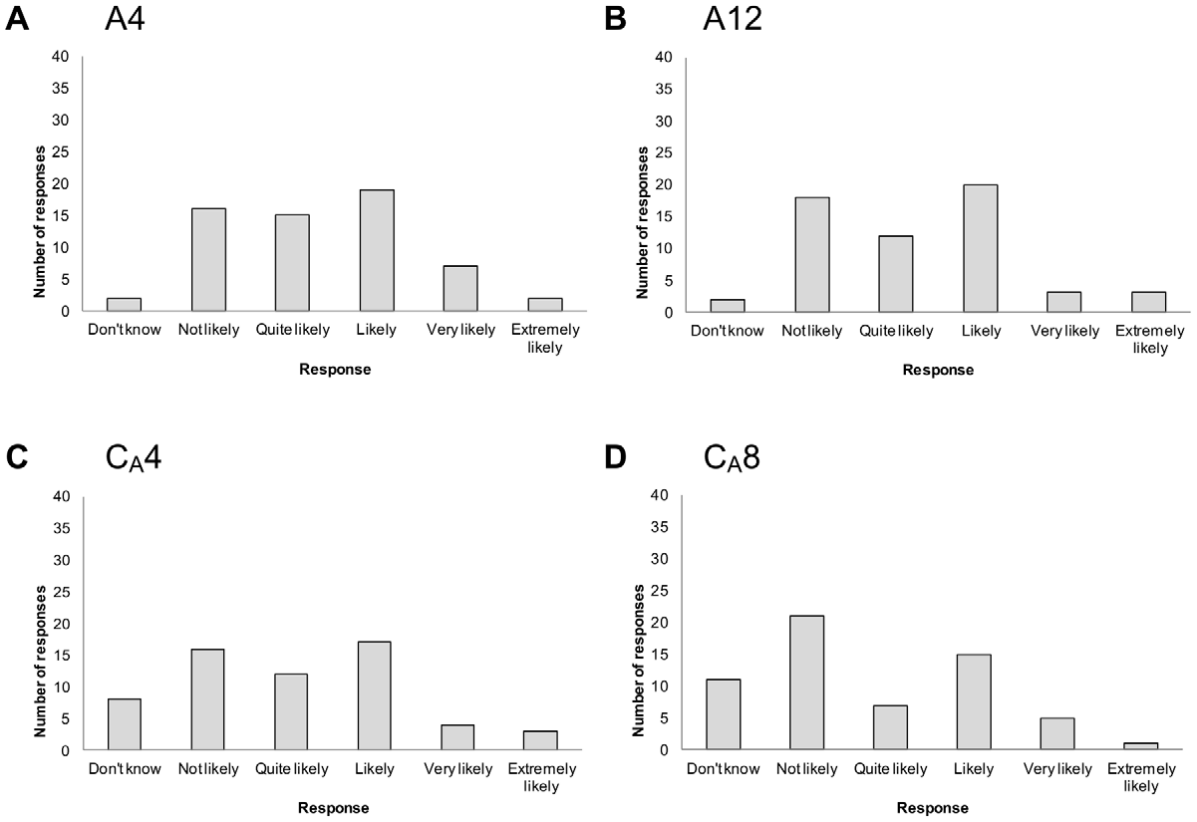
Opinion of 64 experts about the likelihood that ‘*a criterion*’ will influence infectious disease emergence - examples in which participants' opinions were apparently contradictory. Criteria (number and description): a: A4 current incidence of human disease in Canada; b: A12 amount of information that is known about risk factors for introduction and transmission; c: C_A_4 an increase in summer temperature in Canada; d: C_A_8 an increase in summer precipitation in Canada.

The response to a small number of questions could almost be characterised as being ‘bimodal’, in that the two most highly selected responses were ‘not likely’ and ‘likely’. These criteria included current incidence of human disease in Canada, the amount of information that is known about risk factors for introduction and transmission, an increase in summer temperature on air-borne diseases, and an increase in summer precipitation on air-borne diseases (Figure 5a, b, c and d respectively).

There were a total of 12 criteria which at least 20% of participants thought were not likely to influence the probability of disease emergence. Of these, ‘not likely’ was the modal answer in 7 cases. All of these criteria related to the influence of climate change. The criteria were: a decrease in summer temperature (all modes of transmission) a decrease in winter temperature (all modes of transmission) and a decrease in winter precipitation (all modes of transmission), a decrease in summer precipitation (food & water-borne, air-borne and direct/indirect contact), an increase in winter precipitation (air-borne and direct/indirect contact), an increase in winter temperature (air-borne) and an increase in summer precipitation (air-borne and direct/indirect contact). However for all of these criteria, there were always some participants who indicated that this criterion was likely to influence disease emergence. None of the participants suggested removing any of the criteria.

### Uncertainty in expert opinion

To summarise uncertainty the percentage of ‘don't know’ responses out of the total responses to all criteria within a group was calculated. The highest levels of uncertainty were associated with the influence of climate change on air-borne and on direct/indirect contact diseases. Group percentages were as follows (numbers in brackets show the percentage, followed by the minimum and the maximum number of ‘don't know’ responses for criteria in the group: [A] disease epidemiology (3.0, 0–5), [B] ability to monitor, treat and control disease (0.8, 0–1), [C_V_] influence of climate change on vector-borne disease (5.1, 0–8), [C_FW_] food and water-borne disease (5.1, 1–5), [C_A_] air-borne disease (19.8, 6–13), [C_D_] direct/indirect contact diseases (20.7, 5–12), [D] burden of disease (1.7, 0–2) and [E] economic, environmental and social impact (0.6, 0–1).

No more than 10% of experts selected ‘don't know’ in response to any of the criteria in sections A, B, D or E. In section C no more than 10% selected ‘don't know’ for food and water-borne diseases. More than 10% of responses were ‘don't know’ to two criteria relating to vector-borne diseases (an increase in winter precipitation and a decrease in winter precipitation). More than 10% of responses were ‘don't know’ for 9 (of 11) criteria for air-borne disease and 10 (of 11) criteria for direct/indirect contact disease. More than 20% of responses were ‘don't know’ for only one criterion (a decrease in winter precipitation for air-borne disease). There were no criteria for which ‘don't know’ was the modal response.

### Expert suggestions

Participants were invited to comment on any aspect of the questionnaire as they felt appropriate. Some participants suggested additional criteria as follows:

- Presence of reservoir species and intermediate host species.
- Species susceptibility (including vectors).
- Number of potential hosts that can carry the pathogen.
- Likely incidence of disease in wild animals.
- Pathogen infectivity (ease of spread).
- Frequency of contacts with the pathogen (e.g. air travel routes, importation of goods).
- Probability of an outbreak in humans and probability of an outbreak in animals.
- Notifiable status of the disease in Canada at the provincial, territorial or federal levels.
- Impact on animal welfare, public health, international trade and wider society (which includes environment and media).
- Potential impact on food security of First Nations, Inuit and Metis people.
- Potential impact on development of diagnostic techniques for northern diseases.
- Ability of the pathogen to survive in environmental media outside of the host (e.g. water, soil).
- Other climatic factors: the number of frost free days, humidity (in particular for air-borne diseases), prevailing wind conditions and climatic patterns over time e.g. rainfall patterns.

In addition, some participants suggested criteria that tended to relate to the indirect effects of climate change. Suggestions included: potential socioeconomic and demographic changes in populations, population density, overcrowding, heat stress, flood risk, hygiene, living conditions, and increased use of air conditioning units (as a result of increased temperature).

More general comments noted the complexity and uncertainty associated with prioritisation and highlighted that although climate change will have an effect, the system is very complex and that impacts are difficult to predict. Finally participants suggested focusing on particular issues, for example the speciality of the user (e.g. public health, veterinarian), a specific geographic region of Canada, or a specific disease group.

## Discussion

### Expert response rate and revision of responses

In order to design a reliable method of disease prioritisation, criteria need to be explicit and should minimise the influence of factors such as personal interest and political agenda [17]. This work therefore invited input from a number of experts in order to account for a range of opinions and to follow recommendations that criteria should be open to criticism and revision (e.g. [17], [23], [24]). Other disease prioritisation work has rarely used such a large panel of experts (for a review see [23]). A large group has the advantage of providing a substantial sample size, although may be a disadvantage if those individuals have varying agendas and are unable to reach consensus. Conversely, a small number of experts are more likely to provide a biased set of answers reflecting the perspectives of, for example, one particular sector or country of origin [25]. Attempts were made to mitigate such bias by including individuals from a range of backgrounds and by inviting participants to revise their answers as an aid to reaching consensus. Although the response rate to the summary of group results only reached 30%, this is comparable to other studies that have assessed disease risks using a Delphi approach, either remotely via email or post [26], [27], or face to face at a conference [28]. Lack of response has been attributed to the repetitive and time consuming nature of the procedure. In this study a Delphi approach would more appropriately have involved a group meeting, however this was not possible due to limited resources and the wide geographic spread of the participants. It is important to note however, that group meetings also have limitations; in particular they must avoid the group being dominated by shared knowledge or over-strong opinions and particularly the tendency of the group towards overconfidence [21]. It is for these reasons that other expert elicitation studies, for example, to assess food-borne disease in Canada [29] or to evaluate control strategies of contagious animals diseases [26], [30] have preferred to elicit opinion via written questionnaire to prevent any interaction or exchange of information between participants.

The response rate did not vary according to sector and participants tended to decline due to lack of time. Expert input was not weighted according to sector because the background information available (e.g. professional affiliation, geographic location) was not considered to be a reliable way to assess bias. Academic researchers based at universities, for example, are often involved in government advisory roles and could not be classed as purely ‘academic’. One study which did weight experts according to their level of expertise in relation to specific pathogens found that this had little effect on the overall conclusions [31]. The authors would recommend collecting information about expert affiliation during the elicitation phase.

This work assessed a relatively large number of criteria compared to other disease prioritisation work [10], [11], [14], [15], [17], [18], [32], (the maximum found in the literature was forty [11]). The most recent prioritisation in Canada established priorities for national communicable disease surveillance in 1998 using only ten criteria [17]. In general these prioritisation exercises dealt with disease emergence and did not specifically consider climate change.

### Criteria ranking

Criteria were ranked according to a mean score from the expert opinion as a way to highlight those of most concern. The rank of a criteria is likely to depend on the method of scoring and on the measure used to rank (e.g. mean score) and therefore future work to build a prioritisation tool will consider alternative methods of ranking. Sensitivity analysis was conducted here in order to assess the influence of uncertainty i.e. a ‘don't know’ score on the ranking. When the rank was recalculated as the mean score including values of 0, there was little influence on overall ranking. The top 15 criteria remained in the top 15 (only three criteria changed rank) and of the lowest 15 ranked criteria, only 2 were different to the original ranking method. This is primarily a reflection of the limited proportion of ‘don't know’ responses overall, as well as their relatively homogeneous spread across criteria.

### Summary of expert opinion

The majority (six) of the ten most highly ranked criteria were related to potential impacts of any emerging infectious disease on the human population. After the economic impact, the severity, fatality and likely incidence in the human population were of most concern.

#### Disease epidemiology

Experts agreed that disease epidemiology (in particular the mode of transmission and the type of climate that the pathogen can tolerate) is likely to influence disease emergence. Taxonomic group and zoonotic potential were both ‘very likely’ to influence disease emergence, although there was considerable variation between experts despite evidence that the majority of emerging disease events are zoonotic and are caused by bacteria including rickettsia [16], [33], [34]. Methods of pathogen entry were also likely to influence pathogen emergence and a number of high risk methods of pathogen introduction have been identified e.g. via human immigration, import of animal or animal products [35] and via wild bird migration [36], [37].

#### Ability to monitor, treat and control disease

Effective surveillance was highlighted as a priority for infectious disease control (rather than diagnosis, prevention or treatment measures) as it has been in other studies [38]. The other measures are more likely to be employed once the pathogen has emerged.

#### Influence of climate

The climate criteria that ranked most highly (type of climate that the pathogen can tolerate and the current climatic conditions in Canada (in relation to vector-borne diseases) indicate that experts were most concerned about whether the current climate would support pathogen survival and transmission. Participants' overall responses were in agreement with the current literature which indicates that vector-borne diseases are most likely to be influenced by the direct effects of climate change, followed by food and water-borne diseases [1]. This is unsurprising because diseases that are most likely to respond to climate change are those where the pathogen spends some time in the environment. Annual and seasonal increases in temperature and an increase in summer precipitation were deemed most influential. Indeed, it is well known that increasing temperature and precipitation tends to promote disease incidence because the period of time required for pathogen replication decreases and because blood-feeding arthropods often increase their biting frequency and replication rates [39]. Similarly, the risk of enteric disease is likely to increase with increasing temperature and precipitation [5], [40], [41]. Water-borne outbreaks in Canada have been associated with heavy precipitation, snowmelt and flooding [42], [43]. Higher temperatures in the Canadian Arctic have implications for traditional lifestyles, wild food availability and food storage, and may therefore result in an increase in food-borne diseases such as gastroenteritis and botulism [8].

Participants were less concerned and also less certain about the influence of climate on air-borne and direct/indirect contact pathogens. Evidence for the influence of climate compared to other modes of transmission may not be as abundant; however climate does have known effects. A warmer and wetter winter may reduce the occurrence of communicable diseases such as influenza, while warm, dry summers and heavy precipitation in winter provide optimal conditions for transmission of fungal spores that cause, for example, *Coccidioides immitis* [1]. Participants appeared to reflect this understanding, by judging that annual and seasonal increases in temperature and increases in summer precipitation would be most influential. However, participants did not appear to agree that a decrease in summer precipitation would be more influential than an increase in summer precipitation on air-borne transmission.

#### Burden of disease

The burden of disease was a high priority, with all four criteria relating to human disease featuring in the top 10. In comparison the burden in the animal population was of considerably less concern. At least two experts commented that policymakers will always prioritise human health while animal health concerns will be secondary and will typically be measured in terms of loss of trade.

#### Economic, environmental and social impact

The expert opinion supports other work that has stressed the need to consider economic, social and environmental impact either within the same prioritisation or separately [44], since impact of emergence can have considerable and long-lasting consequences (e.g. [45], [46]).

### Expert suggestions

Participants suggested additional criteria for prioritisation purposes. Many were already addressed in almost equivalent terms within the questionnaire. For example, suggestions of ‘probability of an outbreak’ or ‘frequency of contacts with the pathogen’ were addressed with criteria such as ‘number of ways that the pathogen can enter Canada’ and ‘likely incidence of disease in humans or animals’. One suggestion, which was not covered in this assessment, and might be considered in future, was the role of wildlife in pathogen emergence. There were a number of suggestions for additional criteria relating to disease impact, however as the purpose of this work was not a full risk assessment only a few ‘general’ impact criteria were included.

Additional climate criteria suggestions (the influence of wind or extreme events such as floods or droughts, and changes in climate over time), while undoubtedly influential, might prove difficult to estimate for many diseases in the Canadian context, particularly in a timely and reliable manner as required for the tools that will be developed from this work. Other suggestions relating to the indirect influence of climate on disease were also excluded due to the uncertainty and complexity of the subject [6]. One participant summed up this perspective: “changes in vector biology may or may not be overridden by changes in global movement patterns of humans which may or may not be impacted by human encroachment on wildlife, which may or may not be affected by improvements in living conditions of some people in some places as a result of predicted climate change”.

The criteria used here were relatively simple. However, the ultimate aim of this research is to design a practical method of disease prioritisation which can identify pathogens that are likely to be influenced by climate compared to those that are unlikely to be influenced by climate. The criteria focused on the direct influence of climate on the pathogen rather than on the disease and asked separate questions about the availability of the host and vectors, rather than assessing factors related to the pathogen, disease and host in a single question. This is an important point, since the influence of climate on the pathogen may be different to its influence on the disease. For example, an increase in dry climate in western Canada could result in a decline in cattle, other livestock or some wildlife species, and therefore the emergence of a disease could be impacted, although the effect on the pathogen might be negligible.

The importance of qualitative assessment (such as this) in developing strategies to adapt to climate change has been stressed in a number of studies [31], [47]. One recent assessment, for example, of the impact of climate change on vector-borne viruses in the European Union through the elicitation of expert opinion also focused on temperature and precipitation after noting that quantitative information is limited [31]. Although quantitative studies on the burden of disease attributable to climate change are currently scarce [48], measurement of disease burden using Disability Adjusted Life Years (DALYs) have been developed [49]. While this requires assessment in different populations and different ecological regions, incorporation of such measures in prioritisation models would be advantageous where possible.

### Variability

There were some criteria where expert opinion varied considerably. It is likely that this was the result of disagreement between experts. It is also possible that some criterion were not specific enough, or had relevance only to certain pathogens. It is unlikely to be due to lack of knowledge or uncertainty since in these cases experts would have chosen to answer ‘don't know’. The low response rate to the summary of group results was disappointing and it is possible that a higher response rate may have resolved some of the variation in opinion. However, it is worth noting that only 4 out of 19 experts chose to alter their opinion and that a greater response rate might not necessarily result in agreement. While lack of consensus does raise questions as to whether such criteria should be included, it is reasonable to include them (perhaps with a lower weighting) because at least some experts deemed them influential. The variation in response does not necessarily need to be considered as of concern. Diversity of expert views itself carries valuable information [12], and the variation in response can be accounted for in a practical manner when designing a decision support tool.

The variation in response is unlikely due to difference in interpretation of the ‘likelihood’ and ‘importance’ qualitative scoring system even though experts were not provided with a definition for each description. This is for a number of reasons. Phrases were ordered on a five-tiered Likert scale according to their meaning and no description or numerical values were attached. Experts therefore chose an option relative to the other options on the scale. Other studies have used a similar method of qualitative scoring that have allowed consistent expert interpretation, e.g. a likelihood scale [50], a low to high scale [31], or a scale from 1 to 10 [51]. None of the participants in the pilot study nor the experts requested definitions for the likelihood and importance scale.

A qualitative scale was deemed most appropriate for this study, based on the fact that individuals prefer to use imprecise methods such as verbal description of uncertainty when applied to events in which the nature of the underlying uncertainty is also vague compared to numerical responses when representing aleatory uncertainty (chance or probability) [52]. Furthermore, qualitative approaches tend to take less time than a quantitative approach.

### Uncertainty

There appeared to be uncertainty about some of the criteria, which was reflected by the number of experts who responded with ‘don't know’. The greatest amount of uncertainty related to the influence of climate on air-borne and direct/indirect contact diseases. It is likely that this is due to limited availability of information about predictable changes in climate, the influence of climate on pathogens as discussed above or lack of reliable information about the likely drivers of pathogen emergence in the Canadian context, in particular for diseases that are currently exotic. Uncertainty may also result from the level of expertise of individual participants. Although some work has shown that uncertainty was not necessarily due to the self-attributed level of expertise [29], in hindsight it may have been useful to ask experts to indicate their level of certainty about each criterion during the elicitation process. This was not done because it was considered to be too time consuming and would likely reduce the response rate. It is also important to note that it is doubtful whether an expert's own subjective judgement of their ‘level of certainty’ provides a good estimate of the value of the information or whether it introduces more bias [53]. In this study a number of individuals declined to participate due to a self-assessed lack of expert knowledge and it is therefore possible that individuals were included, to some extent, who were confident in their own experience level (as in [54]).

### Conclusion

Although our study cannot account for the complexity that underlies disease dynamics, it can provide a basis for early warning and identification of potential pathogen threats. Collation of expert opinion provides the foundation for a broad, generic tool for disease prioritisation. Future work will develop ranking methods and multi-criteria decision analysis tools that consider the complex nature of disease prioritisation and that are necessary for a consistent and structured method for prioritising emerging (or re-emerging) infectious diseases associated with climate change.
